# Development and validity of a questionnaire to test the knowledge of primary care personnel regarding nutrition in obese adolescents

**DOI:** 10.1186/1471-2296-14-102

**Published:** 2013-07-18

**Authors:** Lucinéia de Pinho, Paulo Henrique Tolentino Moura, Marise Fagundes Silveira, Ana Cristina Carvalho de Botelho, Antônio Prates Caldeira

**Affiliations:** 1 Health Sciences Postgraduate Program, State University of Montes Claros (Unimontes), Montes Claros, MG, Brazil

**Keywords:** Adolescent nutrition, Questionnaire, Validity, Primary healthcare

## Abstract

**Background:**

In light of its epidemic proportions in developed and developing countries, obesity is considered a serious public health issue. In order to increase knowledge concerning the ability of health care professionals in caring for obese adolescents and adopt more efficient preventive and control measures, a questionnaire was developed and validated to assess non-dietitian health professionals regarding their Knowledge of Nutrition in Obese Adolescents (KNOA).

**Methods:**

The development and evaluation of a questionnaire to assess the knowledge of primary care practitioners with respect to nutrition in obese adolescents was carried out in five phases, as follows: 1) definition of study dimensions 2) development of 42 questions and preliminary evaluation of the questionnaire by a panel of experts; 3) characterization and selection of primary care practitioners (35 dietitians and 265 non-dietitians) and measurement of questionnaire criteria by contrasting the responses of dietitians and non-dietitians; 4) reliability assessment by question exclusion based on item difficulty (too easy and too difficult for non-dietitian practitioners), item discrimination, internal consistency and reproducibility index determination; and 5) scoring the completed questionnaires.

**Results:**

Dietitians obtained higher scores than non-dietitians (Mann–Whitney U test, P < 0.05), confirming the validity of the questionnaire criteria. Items were discriminated by correlating the score for each item with the total score, using a minimum of 0.2 as a correlation coefficient cutoff value. Item difficulty was controlled by excluding questions answered correctly by more than 90% of the non-dietitian subjects (too easy) or by less than 10% of them (too difficult). The final questionnaire contained 26 of the original 42 questions, increasing Cronbach’s α value from 0.788 to 0.807. Test-retest agreement between respondents was classified as good to very good (Kappa test, >0.60).

**Conclusion:**

The KNOA questionnaire developed for primary care practitioners is a valid, consistent and suitable instrument that can be applied over time, making it a promising tool for developing and guiding public health policies.

## Background

In recent years, the worldwide prevalence of overweight and obesity in children and adolescents has increased significantly [1]. The high overweight incidence is considered a serious public health issue in the United States [2] and developing countries [3,4], highlighting its importance for healthcare providers and administrators [5].

Obesity has become an epidemic and, although this situation must be remedied, current public healthcare models have been inefficient in doing so. In order to revert this situation, a number of approaches have been suggested to prevent, evaluate and treat overweight and obesity in children and adolescents [6], particularly in primary care [7]. However, the success of any action plan depends on primary care staff’s having the knowledge and ability to prevent overweight and obesity in children and adolescents. Obese children and teenagers and their families require extensive guidance and assistance to increase their self-management capabilities and promote a healthy lifestyle [4].

The role of primary care practitioners is to provide obese patients with basic dietary counseling and, if necessary, refer them to a dietitian for individual care [8]. However, primary healthcare providers are rarely capable of providing dietary counseling, [8,9]. This is a serious problem since nutrition is essential as auxiliary therapy in the treatment of chronic illnesses [10]. These diseases have increased drastically in recent years and currently affect a significant portion of the population [10].

Against this backdrop, the nutritional counseling provided to obese patients must be improved and standard protocols established [8]. Primary care practitioners can provide effective nutritional counseling if they: 1) receive appropriate training, 2) are given effective tools, 3) operate in an organized system and, 4) are assisted by qualified dieticians [11]. For family physicians, successful implementation of Clinical Practice Guidelines on the Management and Prevention of Obesity requires adequate training, and evidence-based interventions related to nutrition [9].

Primary care practitioners should be trained not only to evaluate overweight and obesity in children and adolescents, but also to provide these patients with the care required for a healthier life. In order to adequately treat these conditions, care providers should participate in specific and continuous training programs [5].

In light of the above, it is important to evaluate the knowledge of primary care practitioners regarding adolescent nutrition and obesity. However, the tools used to assess these disorders in teenagers were developed for patients, and protocols to evaluate the knowledge of non-dietitian primary care practitioners are as yet unavailable. Though scarce, these studies are essential in guiding public health policies developed to combat childhood and adolescent obesity, and in creating training programs that enable primary care practitioners to provide adequate nutritional counseling to overweight and obese children and adolescents.

The present study was conducted to compile and validate a specific questionnaire to test the Knowledge of Nutrition in Obese Adolescents (KNOA) of non-dietitian primary care practitioners.

## Methods

**Figure 1:**
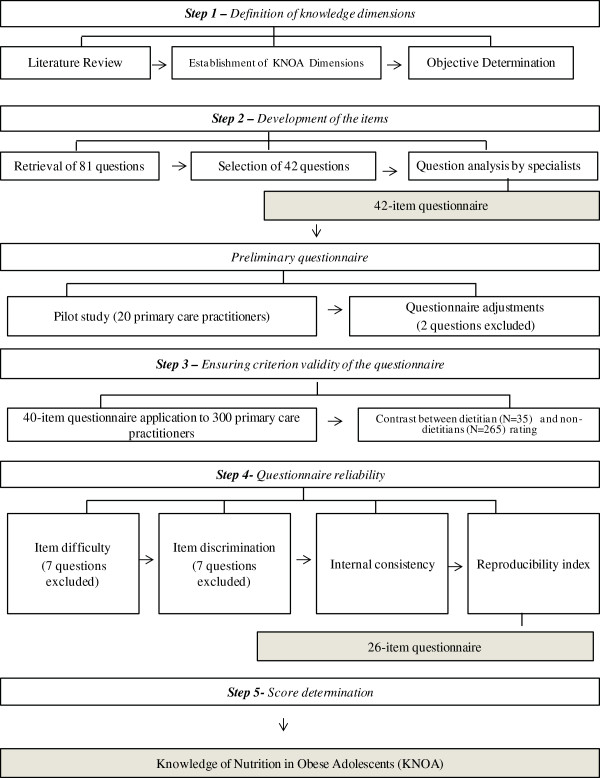
Summary of steps for developing a questionnaire to assess KNOA in non-dietitian health professionals.

The dimensions of knowledge and question were based on the studies listed in Appendix 1. They consist of didactic materials and guidelines for healthy adolescent diets available to primary care practitioners, some of which are recommended by the Brazilian Ministry of Health. The questionnaire development and validation procedure is illustrated in Figure 1 and described below.

### Step 1 – definition of knowledge dimensions

KNOA was considered to be the nutritional knowledge that any primary care practitioner must have in order to treat obese adolescents. After an extensive literature review (Appendix 1) we didactically selected dimensions for KNOA (Table 1), as follows: epidemiology of obesity in adolescence; clinical implications of obesity in adolescence; mapping obesity in adolescence; nutritional management for obese adolescents, and knowledge of fruit, vegetable, fat and sugar consumption, as described in Table 1. Albeit somewhat arbitrary, this categorization was useful in obtaining better balance in the next step of the study.

**Table 1:** Dimensions of knowledge on KNOA and its objectives

**Dimension**	**Objective**
Epidemiology of obesity in adolescence	Understanding the distribution and determinants of obesity in adolescence.
Clinical implications of obesity in adolescence	Association between obesity in adolescence and health loss, diseases and associated complications.
Mapping obesity in adolescence	How obesity is diagnosed in adolescents and how this issue is discussed with the patient.
Nutritional management for obese adolescents	Knowledge on general nutritional measures for treating obese adolescents.
Fruit and vegetable consumption	Knowledge of the nutritional properties of fruits and vegetables, their necessity and importance for obese adolescents.
Fat consumption	Knowledge of the nutritional properties of fats, their requirement in adolescents and adjustments for obese individuals.
Sugar consumption	Knowledge of the nutritional needs of adolescents in terms of carbohydrates and sugars and adjustments for obese individuals, considering their eating habits (food, candy, desserts and sweeteners).

### Step 2 – Development of the items

Considering the studies listed in Appendix 1, we selected 81 sentences related to the KNOA dimensions described in Table 1 (more than 10 questions per dimension). From this database we selected 42 questions based on two criteria: the frequency with which the issues arose in the different studies and their importance and prevalence in clinical practice. The initial questionnaire was elaborated and applied in Portuguese.

This initial questionnaire was submitted to a panel of five experts: two dietitians, two pediatricians and one nurse, who evaluated the appropriateness, relevance, accuracy, and formulation of each question. The adequacy and clarity of the questionnaire was also assessed in a pilot study conducted with 20 primary care practitioners (2 physicians, 13 nurses and 5 dentists) belonging to a private health group in a city in northern Minas Gerais state. The pilot study was performed to check for clarity and readability of individual questionnaire items and the adequacy of this tool in assessing the knowledge of health practitioners, thereby contributing to the face validity of the questionnaire. Problems raised by the health professionals were noted, and suitable changes were made accordingly. Unclear and dubious questions were excluded.

### Step 3 – Ensuring criterion validity of the questionnaire

To determine criterion validity, all 402 primary care practitioners registered with the Secretary of Health in a city in northern Minas Gerais and dietitians from public and private services were invited to complete the 42-item questionnaire. All the subjects had at least two years of primary care experience. They provided personal information (demographic and educational) and were also questioned regarding the care of obese adolescents, who took the initiative to talk about nutrition (practitioner or patient), and the main barriers to discussing this subject.

The professionals were expected to achieve a high or low rate of correct answers in accordance with their level of nutritional education [12]. Thus, dietitians were expected to achieve a higher mean score than primary care practitioners, with a statistically significant difference to ensure criterion validity. The questionnaire was applied at their workplace in the first half of 2012.

### Step 4 – Questionnaire reliability

Several procedures were implemented to increase questionnaire reliability:

a) in validity testing, items correctly answered by more than 90% of the 265 non-dietitians (considered too easy) or less than 10% (considered too difficult) were excluded [13];

b) items were discriminated by correlating the score of each item with the mean score of the complete questionnaire. A minimum cutoff point of 0.2 was used to determine the correlation coefficient between the total mean score and each questionnaire item, and items with lower values were excluded [14];

c) the internal consistency (reliability) of questionnaire results was evaluated using Cronbach α values [15];

d) the reproducibility index was assessed over two weeks, applying the reformulated version to nearly 20% (60 subjects) of the initial sample [16].

### Step 5 – Score determination

A score ranging from -1 to +1 was determined for each dimension. Items were scored -1 for an incorrect answer, 0 when the response was “I don’t know” and +1 for correct answers. The mean values of the items were calculated to obtain the dimension scores for each respondent, followed by calculation of the mean score for each group.

#### ***Data analysis***

Descriptive statistics with distribution frequency was used to characterize the sample.

The Mann–Whitney U test was applied to compare dietitians with non-dietitians in questionnaire validity testing (Phase 3), using a significance level of P < 0.05.

In Step 4, the *Kappa* test was used to evaluate agreement between mean test-retest scores for questionnaire items [16].

Statistical tests were carried out using *Statistical Package for the Social Science* (SPSS) software, version 15.0.

#### ***Ethical questions***

The investigation respected ethical guidelines for research with human beings. Participation was voluntary and subjects gave their informed consent. The study was approved by the Research Ethics Committee of the *Universidade Estadual de Montes Claros*, under protocol number 3016/2011.

## Results

### Questionnaire development and validity

#### ***Subject characterization***

Thirty five dietitians and 265 non-dietitian primary care practitioners (76 physicians, 139 nurses and 50 dentists) participated in the study. Most participants were women (65%), aged 32.7 ± 8.6 years, who had graduated less than 5 years before (54%), and were specialized in primary health care (67%). A total of 84.3% of participating practitioners treated obese adolescents. Among the main difficulties in caring for these patients were poor patient adherence to recommendations (41.3%) and lack of knowledge regarding adolescent nutrition (25.4%) (Table 2).

**Table 2:** Demographic characteristics and experience of participants in the questionnaire validity test

**Variables**	**Dietitians (n = 35)**	**Non-dietitians (n = 265)**	**Total (n = 300)**
	**N (%)**	**N (%)**	**N (%)**
**Gender**			
Female	32(91.4)	165(62.3)	197(65.7)
Male	3(8.6)	100(37.7)	103(34.3)
**Age (years)**			
≤ 25	10(28.6)	30(11.3)	40(13.3)
26-35	22(62.8)	177(66.8)	199(66.3)
36-45	3(8.6)	31(11.7)	34(11.3)
46-55	0	13(4.9)	13(4.4)
≥ 56	0	14(5.3)	14(4.7)
**Time since graduation**			
≤ 5 years	20 (57.1)	142(53.6)	162(54)
> 5 years	15(42.9)	123(46.4)	138(46)
**Specialization**			
Yes	20(57.1)	180(67.9)	200(66.7)
No	15 (42.9)	85(32.1)	100(33.3)
**Treated obese adolescents**			
Yes	26(74.3)	227(85.7)	253(84.3)
No	9(25.7)	38(14.3)	47(15.7)
**Barriers to discuss “Nutrition”**			
No barriers	10(28.6)	44(16.6)	54(18)
Short consultation time	7 (20.0)	39(14.7)	46(15.3)
Non-adhesion to the treatment	11(31.4)	113(42.7)	124(41.3)
Lack of knowledge	7(20.0)	69(26.0)	76(25.4)

#### ***Final questionnaire***

Seven of the initial 42 questions (Step 4) were excluded by the difficulty criterion: 7 based on the low correlation obtained in item discrimination and 2 after the pilot study because they were considered too specific for a questionnaire involving non-dietitian professionals. The final questionnaire contained 26 items (Table 3).

**Table 3:** Final version of the KNOA questionnaire for primary care practitioners

**Questions**	
1 –	Most studies indicate that the prevalence of overweight or obesity in adolescents ranges from 10% to 15%.
2 –	Changes in nutrition habits such as increase in carbohydrate and fat consumption are directly associated to the current prevalence of adolescent obesity.
3 –	Obese adolescents have potential for becoming obese adults.
4 –	The chance of obese adolescents developing type 2 diabetes is 2 to 3 times greater than that of non-obese adolescents.
5 –	At least 10% of obese adolescents present with arterial hypertension.
6 –	Obesity in adolescents is positively related to dyslipidemia.
7 –	Body mass index (BMI) is considered a sufficient indicator of the nutritional status of adolescents.
8 –	Preceding the pubertal growth spurt, adolescents may exhibit an overweight appearance that is not diagnosed as obesity.
9 –	Adolescents with a weight-for-age percentile greater than 85% are diagnosed as obese.
10 –	Adolescents undergoing treatment must be evaluated every 6 months.
11 –	In the treatment of obese adolescents, weight gain interruption, with weight stabilization within a growth chart percentile which represents obesity can be assumed to be a satisfactory preliminary therapeutic result.
12 –	Food guide pyramids should be shown to patients to explain nutrient variety, moderate consumption and proportion of food items.
13 –	It is recommended that obese adolescents have 4 daily meals: breakfast, lunch, afternoon snack and dinner.
14 –	Fruit can be replaced by fruit juice.
15 –	The health benefits of fruits and vegetables are that of providing vitamins and mineral salts.
16 –	Consuming one apple and one banana every day meets daily fruit consumption recommendations.
17 –	Consuming fiber-rich fruits promotes a feeling of satiety, contributing to weight control.
18 –	Fats must be excluded from the diets of obese adolescents.
19 –	Obese adolescents should consume “diet food” to limit dietary fat.
20 –	Obese adolescents should avoid drinking milk due to its high fat content.
21 –	Obese adolescents may include low-fat sandwiches (containing turkey breast, ricotta cheese and green leaves) in their diet.
22 –	In contrast to saturated fats, unsaturated fats do not cause health problems unless they are consumed excessively.
23 –	The amount of sweets or sugary food items recommended for obese adolescents is limited to a maximum of one daily portion.
24 –	The use of artificial sweeteners as a substitute for sugar is indicated in the treatment of obese adolescents.
25 –	Carbohydrates with a low-glycemic index are known to play a positive role in dietotherapy of obesity.
26 –	Fruit-based rather than creamy desserts should be adopted when treating obese adolescents.

#### ***Criterion validity of the questionnaire***

As expected, the mean score obtained in the dimensions evaluated was higher for dietitians than non-dietitians, except for dimension I – Epidemiology of obesity in adolescence (Table 4).

**Table 4:** Mean score of primary care practitioners on the KNOA questionnaire

**Dimensions**	**Dietitians (n = 35)**		**Non-dietitians (n = 265)**		p*
	**Mean (DP)**	**Median**	**Mean (DP)**	**Median**	
I	0.419(0.356)	0.333	0.367(0.440)	0.333	0.609
II	0.800(0.335)	1.000	0.646(0.443)	0.667	0.035
III	0.600(0.403)	0.333	0.252(0.517)	0.333	0.000
IV	0.507(0.418)	0.500	-0.012(0.476)	0.000	0.000
V	0.371(0.475)	0.500	-0.056(0.550)	0.000	0.000
VI	0.709(0.341)	0.800	0.219(0.512)	0.200	0.000
VII	0.571(0.306)	0.500	0.199(0.438)	0.250	0.000
Total	0.569(0.184)	0.615	0.208(0.314)	0.230	0.000

*Statistical different between the groups (Mann–Whitney U test).

Based on score distribution into quartiles (Q1, Q2, Q3, Q4), the KNOA of both groups was categorized as insufficient (lower than Q1: KNOA < 0.0385); fair (between Q1and Q2: 0.0386 ≤ KNOA ≤ 0.2790); good (between Q2 and Q3: 0.2800 ≤ KNOA ≤ 0.4590) and very good (higher than Q4: KNOA ≥ 0.4600). This classification showed that 94% of dietitians exhibited good to very good KNOA, while 55% of non-dietitian respondents exhibited fair or insufficient KNOA (Table 5).

**Table 5:** Classification of KNOA in primary care practitioners

**KNOA**	**Dietitians (n = 35)**		**Non-dietitians (n = 265)**	
	**N**	**%**	**N**	**%**
Insufficient	0	0	80	30.2
Fair	2	5.7	68	25.7
Good	5	14.3	57	21.5
Very good	28	80.0	60	22.6

*KNOA* Knowledge of Nutrition of Obese Adolescents.

#### ***Internal consistency***

Principal Component Analysis was applied to the knowledge dimensions (Table 1), and since the results did not support a factor structure, the questionnaire was treated as a single scale. Accordingly, a single Chronbach α value of 0.788 was calculated for the original 42-item questionnaire. Following the exclusion of 16 inadequate items, the α value increased to 0.807, indicating satisfactory internal consistency.

Test-retest agreement between answers (Phase 4) was classified as good and very good (Table 6).

**Table 6:** **Reproducibility of responses to the KNOA questionnaire determined by the *Kappa *test**

Kappa value*	**Items**
0.60-0.70	5,9,10,24,26
0.71-0.80	2,4,6,14,16,18,19,21,23,25
0.81-0.9	1,3,11,12,13,15,20,22
> 0.91	7,8,17

* Classification for Kappa’s agreement test: very good = 0.81-1.00; good = 0.61-0.80; moderate = 0.41-0.60; fair = 0.21-0.40 and poor ≤ 0.20.

## Discussion

The present study met the goal of developing a questionnaire to test the Knowledge of Nutrition in Obese Adolescents (KNOA) among non-dietitian primary care practitioners. Other studies have validated tools assessing the eating behavior of adolescents, but these were not designed for healthcare practitioners.

Johnson *et al*., (2002) compiled and validated a 23-item checklist to measure eating behavior in adolescents by analyzing fat, fiber, fruit and vegetable consumption, dietary restrictions, knowledge on nutrition and family income. Turconi *et al.*[17] developed a questionnaire on the food habits, eating behavior and nutritional knowledge of teenagers, with potential for measuring the effects of nutritional interventions. Whati *et al*. [13] designed and validated a survey to assess the nutritional knowledge of South African adolescents aged 13 to18 years. Kruseman *et al*. [18] proposed a self-administered questionnaire for 9 to 15-year-olds to assess their knowledge of nutrition, and which could also be used by primary care practitioners involved in nutritional counseling. To complement these investigations, the present study proposes and validates a tool to assess KNOA in primary care practitioners. According to the data recorded, 84.3% of the primary care practitioners participating in the study had previously treated obese adolescents, reinforcing the presence of these patients in primary health centers as well as the importance of applying the questionnaire to establish strategies that improve their nutritional counseling.

In regard to questionnaire validity, dietitians obtained higher KNOA scores than other practitioners, which was expected considering their educational level. The poor performance exhibited by the other professionals assessed suggests limited KNOA [7,8,10,11]. In practice, this result is relevant and alarming since such a lack of preparation can compromise the nutritional counseling of obese adolescents.

The final version of the questionnaire demonstrated satisfactory internal consistency and reliability, as shown by the Cronbach statistics and test-retest procedure. In addition, questionnaire dimensions must be evaluated concurrently, given that internal consistency decreases when these are analyzed individually. Since the Cronbach α value indicates a positive association with the number of items on a scale, a higher number of questions is associated to greater consistency (Hair et al., 2005). Similar results were obtained in research performed by Steyn *et al*. [14].

The test-retest results indicate temporal stability, allowing comparisons to be drawn over time [17]. Thus, the questionnaire developed can be potentially applied usjng a temporal approach to evaluate, for example, the effects of interventions such as educational programs and training of primary care practitioners.

This study was conducted using intentional sampling of primary care practitioners from a single municipality, and given the cultural particularities of this group, extrapolations of the findings are limited. Thus, questionnaire items related to epidemiology should be adapted or replaced if applied to other localities. Despite this, the present study can gives rise to further investigations on KNOA in primary care practitioners in light of the scarcity of national and international research on this topic. Future studies will explore the potentialities of the questionnaire, considering its use in assessing the effectiveness of educational programs, and diagnosing KNOA problems in primary care practitioners.

## Conclusions

The KNOA questionnaire developed specifically for primary care practitioners is valid, consistent and suitable for application over time in the study area. Although it must be adjusted to be applied to localities of different epidemiological contexts, it is a promising tool in the development and guidance of public health actions related to obesity in adolescence.

## Appendix 1

References used to elaborate the questionnaire on knowledge on KNOA.

1. Aller EE, Abete I, Astrup A, Martinez JA, van Baak MA: **Starches, sugars and obesity.***Nutrients* 2011, **3**: 341–369.

2. Alsaffar AA: **Validation of a general nutrition knowledge questionnaire in a Turkish student sample.***Public Health Nutr* 2012, **15**:2074–2085.

3. Amuna P, Zotor FB: **Epidemiological and nutrition transition in developing countries: impact on human health and development.***Proc Nutr Soc* 2008, **67**:82–90.

4. Anderson AS, Bell A, Adamson A, Moynihan P: **A questionnaire assessment of nutrition knowledge – validity and reliability issues**. *Public Health Nutr* 2002, **5**:497–503.

5. Bibiloni MDM, Martínez E, Llull R, Pons A, Tur JA: **Western and Mediterranean dietary patterns among Balearic Islands’ adolescents: socio-economic and lifestyle determinants*.****Public Health Nutr* 2011, **15**: 683–692.

6. Byrd-Bredbenner C: **A nutrition knowledge test for nutrition educators.***J Nutr Educ* 1981, **13**:97–99.

7. Coordenação Geral da Política de Alimentação e Nutrição: *Alimentação saudável para adolescentes: siga os 10 passos.* Brasília: Ministério da Saúde (Brazil); [http://189.28.128.100/nutricao/docs/geral/10passosAdolescentes.pdf]. (Poster in Portuguese)

8. Crowley J, Ball L, Wall C, Leveritt M: **Nutrition beyond drugs and devices: a review of the approaches to enhance the capacity of nutrition care provision by general practitioners.***Aust J Prim Health* 2012, **18**:90–95.

9. Daniels SR, Arnett DK, Eckel RH, Gidding SS, Hayman LL, Kumanyika S, Robinson TN, Scott BJ, StJeor S, Williams CL: **Overweight in children and adolescents: pathophysiology, consequences, prevention, and treatment.***Circulation* 2005, **111**:1999–2012.

10.  Departamento de Nutrologia: *Obesidade na infância e adolescência: manual de orientação*. São Paulo: Sociedade Brasileira de Pediatria; 2008. (in Portuguese)

11.  Dickson-Spillmann M, Siegrist M, Keller C: **Development and validation of a short, consumer-oriented nutrition knowledge questionnaire.***Appetite* 2011, **56**:617–620.

12.  Dickson-Spillmann M, Siegrist M: **Consumers’ knowledge of healthy diets and its correlation with dietary behaviour.***J Hum Nutr Diet* 2011, **24**:54–60.

13.  Diretoria de Pesquisas Coordenação de Trabalho e Rendimento: **Antropometria e estado nutricional de crianças, adolescentes e adultos no Brasil**. In *Pesquisa de Orçamentos Familiares 2008–2009*. Rio de Janeiro: IBGE/Ministério do Planejamento, Orçamento e Gestão; 2010. (in Portuguese)

14.  Duncan S, Duncan EK, Fernandes RA, Buonani C, Bastos KD, Segatto AF, Codogno JS, Gomes IC, Freitas IF: **Modifiable risk factors for overweight and obesity in children and adolescents from São Paulo, Brazil.***BMC Public Health* 2011, **11**:585.

15.  Eaton CB, McBride PE, Gans KA, Underbakke GL: **Teaching nutrition skills to primary care practitioners.***J Nutr* 2003, **133**(Suppl):563S-566S.

16.  Gidding SS, Dennison BA, Birch LL, Daniels SR, Gillman MW, Lichtenstein AH, Rattay KT, Steinberger J, Stettler N, Van Horn L: **Dietary recommendations for children and adolescents: a guide for practitioners: consensus statement from the American Heart Association.***Circulation* 2005, **112**:2061–2075.

17.  Gidding SS, Dennison BA, Birch LL, Daniels SR, Gilman MW, Lichtenstein AH, Rattay KT, Steinberger J, Stettler N, Horn LV: **Dietary recommendations for children and adolescents: a guide for practitioners.***Pediatrics* 2006, **117**:544–559.

18.  Gomes LMX, Vieira MM, Reis TC, Barbosa TLA, Caldeira AP: **Knowledge of family health program practitioners in Brazil about sickle cell disease: a descriptive, cross-sectional study.***BMC Family Practice* 2011, **12**:89.

19.  Guadagnin SC: **Elaboração e validação de questionários de conhecimentos em nutrição para adultos.***MSc dissertation*. University of Brasília, Human Nutrition; 2010. (in Portuguese)

20.  Gupta N, Goel K, Shah P, Misra A: **Childhood obesity in developing countries: epidemiology, determinants, and prevention.***Endocr Rev* 2012, **33**:48–70.

21.  Hu FB: **Dietary pattern analysis: a new direction in nutritional epidemiology.***Curr Opin Lipidol* 2002, **13**:3–9.

22.  Jacobson D, Gance-Cleveland B: **A systematic review of primary healthcare provider education and training using the Chronic Care Model for childhood obesity**. *Obes Rev* 2011, **12**:e244–e256.

23.  Johnson F, Wardle J, Griffith J: **The Adolescent Food Habits Checklist: reliability and validity of a measure of healthy eating behaviour in adolescents**. *Eur J Clin Nutr* (2002) 56, 644–649.

24.  Kolasa KM, Rickett K: **Barriers to providing nutrition counseling cited by physicians: a survey of primary care practitioners.***Nutr Clin Pract* 2010, **25**:502–509.

25.  Kruseman M, Berchtold A, Truan J, Duboule L, Faurie H, Emonet E, Volery M: **Development and validation of a nutritional knowledge questionnaire among 9- to 15-year-old.***Arch Pediatrie* 2012, **19**:456–466. (in French)

26.  Levy RB, Castro IRR, Cardoso LO, Tavares LF, Sardinha LMV, Gomes FS, Costa AWN: **Consumo e comportamento alimentar entre adolescentes brasileiros: Pesquisa Nacional de Saúde do Escolar (PeNSE), 2009**. *Ciênc Saúde Coletiva* 2010, **15**:3085–3097. (in Portuguese)

27.  Lobstein T, Baur L, Uauy R: **Obesity in children and young people: a crisis in public health.***Obes Rev* 2004, **5**(Suppl 1):S4-85.

28.  Lock K, Pomerleau J, Altmann DR, McKee M: **The global burden of disease attributable to low consumption of fruit and vegetables: implications for the global strategy on diet.***Bull World Health Organ* 2005, **83**:100–108.

29.  Magarey A, Watson J, Golley RK, Burrows T, Sutherland R, Mcnaughton SA, Denney-Wilson E, Campbell K, Collins C: **Assessing dietary intake in children and adolescents: considerations and recommendations for obesity research.***Int J Pediatr Obes* 2011, **6**:2–11.

30.  McNaughton SA, Ball K, Mishra GD, Crawford DA: **Dietary patterns of adolescents and risk of obesity and hypertension.***J Nutr* 2008, **138**:364–370.

31.  Ministry of Health: *Food and Nutrition Guidelines for Healthy Children and Young People (Aged 2–18 years): a background paper.* 1st edition. Wellington; 2012.

32.  Mitchell LJ, Macdonald-Wicks L, Capra S: **Nutrition advice in general practice: the role of general practitioners and practice nurses.***Aust J Prim Health* 2011, **17**: 202–208.

33.  Monteiro CB, Cardoso A, d’Abreu HCC, Ribeiro MG, Bouzas I: **Obesidade na adolescência: reflexões e abordagem.***Adolescência* &*Saúde* 2010, **1**:12–18.

34.  Moreno LA, Rodriguez G, Fleta J, Bueno-Lozano M, Lazaro A, Bueno G: **Trends of dietary habits in adolescents.***Crit Rev Food Sci Nutr* 2010, **50**:106–112.

35.  Ogden CL, Carroll MD, Kit BK, Flegal KM: **Prevalence of obesity and trends in body mass index among US children and adolescents, 1999–2010.** JAMA 2012, **17**:583–590.

36.  Onis M, Onyango AW, Borghi E, Siyam A, Nishida C, Siekmann J: **Development of a WHO growth reference for school-aged children and adolescents.***Bull World Health Organ* 2007, **85**:660–667.

37.  Pan American Health Organization: *Doenças crônico-degenerativas e obesidade: estratégia mundial sobre alimentação, atividade física e saúde.* Brasília; 2003. 58p. (in Portuguese)

38.  Parmenter K, Wardle J: **Development of a general nutrition knowledge questionnaire for adults**. *Eur J Clin Nutr* 1999, **53**:298–308.

39.  Parmenter K, Wardle J: **Evaluation and design of nutrition knowledge measures.***J Nutr Educ* 2000, **32**:260–277.

40.  Rasmussen M, Krølner R, Klepp KI, Lytle L, Brug J, Bere E, Due P: **Determinants of fruit and vegetable consumption among children and adolescents: a review of the literature, Part I: quantitative studies.***Int J Behav Nutr Phys Act* 2006, **3**:22.

41.  Robinson GA, Geier M, Rizzolo D, Sedrak M: **Childhood obesity: complications, prevention strategies, treatment.** JAAPA 2011, **24**:58–63.

42.  Roe L, Strong C, Whiteside C, Neil A, Mant D: **Dietary intervention in primary care: validity of the DINE method for diet assessment.***Fam Pract* 1994, **4**:375–381.

43.  Sargent GM, Pilotto LS, Baur LA: **Components of primary care interventions to treat childhood overweight and obesity: a systematic review of effect**. *Obes Rev* 2011, **12**:e219-e235.

44.  Scagliusi FB, Polacow VO, Cordás TA, Coelho D, Alvarenga M, Philippi ST, Lancha-Júnior AH: **Tradução, adaptação e avaliação psicométrica da escala de conhecimento nutricional do *National Health Interview Survey Cancer Epidemiology.****Rev Nutr* 2006, **19**:425–436. (in Portuguese)

45.  Secretaria de Atenção à Saúde, Coordenação-Geral da Política de Alimentação e Nutrição. *Guia alimentar para a população brasileira.* Brasília: Ministério da Saúde, 2005. (in Portuguese)

46.  Secretaria de Atenção à Saúde, Departamento de Ações Programáticas Estratégicas: *Saúde do adolescente: competências e habilidades.* Brasília: Ministério da Saúde (Brazil); 2008. (in Portuguese)

47.  Secretaria de Atenção à Saúde, Departamento de Atenção Básica: *Matriz de ações de alimentação e nutrição na atenção básica de saúde.* Brasília: Ministério da Saúde (Brazil); 2009. [http://bvsms.saude.gov.br/bvs/publicacoes/matriz_alimentacao_nutricao.pdf] (in Portuguese)

48.  Secretaria de Atenção à Saúde, Departamento de Atenção Básica: *Obesidade*. Brasília: Ministério da Saúde (Brazil); 2006. [*Cadernos de Atenção Básica* n. 12, *Série A: Normas e Manuais Técnicos*] (in Portuguese)

49.  Secretaria de Atenção a Saúde: *Orientações para o atendimento à saúde do adolescente: kit menina*. Brasília: Ministério da Saúde (Brazil); 2009. [http://portal.saude.gov.br/portal/arquivos/pdf/prancha_menina.pdf] (in Portuguese)

50.  Secretaria de Atenção a Saúde: *Orientações para o atendimento à saúde do adolescente: kit menino*. Brasília: Ministério da Saúde (Brazil); 2009. [http://portal.saude.gov.br/portal/arquivos/pdf/prancha_menino.pdf] (in Portuguese)

51.  Siti Sabariah B, Zalilah MS, Norlijah O, Normah H, Maznah I, Laily P, Zubaidah J, Sham MK, Zabidi Azhar MH**: Reliability and validity of the instrument used in the helic (healthy lifestyle in children) study of primary school children’s nutrition knowledge, attitude and practice.***Mal J Nutr* 2006, **12**:33–44.

52.  Stang J, Story M (Eds): *Guidelines for Adolescent Nutrition Services.* Minneapolis: Center for Leadership, Education and Training in Maternal and Child Nutrition, Division of Epidemiology and Community Health, School of Public Health, University of Minnesota; 2005.

53.  Steyn NP, Labadarios D, Nel JH, Heidi-Lee R: **Development and validation of a questionnaire to test knowledge and practices of dietitians regarding dietary supplements.***Nutrition* 2005, **21**:51–58.

54.  Talip WA, Steyn NP, Visser M, Charlton KE, Temple N: **Development and validation of a knowledge test for health professionals regarding lifestyle modification.***Nutrition* 2003, **19**:760–766.

55.  Turconi G, Celsa M, Rezzani C, Biino G, Sartirana MA, Roggi C: **Reliability of a dietary questionnaire on food habits, eating behaviour and nutritional knowledge of adolescents**. *Eur J Clin Nutr* 2003, **57**:753–763.

56.  Turconi G, Guarcello M, Maccarini L, Cignoli F, Setti S, Bazzano R, Roggi C: **Eating habits and behaviors, physical activity, nutritional and food safety knowledge and beliefs in an adolescent Italian population.***J Am Coll Nutr* 2008, **27**:31–43.

57.  Venter I: **Construction of a valid and reliable test to determine knowledge on dietary fat of higher-educated young adults.***S Afr J Clin Nutr* 2008, **21**:133–139.

58.  Wardle J, Parmenter K, Waller J: Nutrition knowledge and food intake. *Appetite* 2000, **34**:269–275.

59.  Whati LH, Senekal M, Steyn NP, Nel JH, Lombard C, Norris S: **Development of a reliable and valid nutritional knowledge questionnaire for urban South African adolescents.***Nutrition* 2005, **21**:76–85.

60.  World Health Organization: *Adolescent nutrition: a neglected dimension.* Geneva; 2003. [http://www.who.int/nut/ado.htm.2]

61.  World Health Organization: *Nutrition in adolescence: issues and challenges for the health sector: issues in adolescent health and development.* Geneva; 2005. [http://whqlibdoc.who.int/publications/2005/9241593660_eng.pdf]

62.  World Health Organization: *Physical status: the use and interpretation of anthropometry.* Geneva; 1995 [*Technical Report Series*, 854].

63.  World Health Organization: *Preparation and use of food-based dietary guidelines.* Geneva; 1998. [http://www.fao.org/docrep/x0243e/x0243e00.htm]

64.  Wynn K, Trudeau JD, Taunton K, Gowans M, Scott I: **Nutrition in primary care Current practices, attitudes, and barriers.***Can Fam Physician* 2010; **56**:e109-e116.

## Abbreviations

KNOA: Knowledge on nutrition in obese adolescents.

## Competing interests

The authors declare that they have no competing interests.

## Authors’ contributions

LP participated in study conception and design, execution, data analysis and interpretation, and compiling and revising the manuscript. PHTM participated in study design and execution. ACCB, APC and MFS participated in study conception and design, research supervision, data analysis, interpretation and revision of the manuscript. All authors approved the final version of the manuscript.

## Pre-publication history

The pre-publication history for this paper can be accessed here:

http://www.biomedcentral.com/1471-2296/14/102/prepub
